# Clinical Notes

**Published:** 1890-02

**Authors:** Filipe Ascot De Tortosa


### CLINICAL NOTES.

By Dr. Filipe Ascot de Tortosa.

Translated from *El Consultor Homœopatico*, No. 24, of Barcelona, Spain, Aug., 1889, by E. A. P.

Miguel Esteller y Castell, aged thirty-one years, of San Jorge, province of Castellon de la Plana, of good constitution, sanguine nervous temperament, had during the past civil war received a gun-shot wound in the right arm which was seemingly cured, without any futher consequences. After three years there appeared on the elbow a small tumor that he tried to cure by emollients first, and after with salves of an action more or less astringent, with some relief, but it reappeared later in the same condition.

He consulted the doctor residing in his town, who treated it with Carbolic acid diluted with water, by injections, and concentrated outward applications of equal strength, but the result was not equal to expectation; on the contrary the tumor ulcerated; kept growing larger and deeper, finding its way into the tendons, suppuration becoming more and more profuse, and eating its way into the anterior portion of the fore-arm, inferior part across bunch of tendons of the abductor and of the extensor-pollicis, leàving uncovered the radius bone a centimetre and a half above the wrist.

He continued thus for some time under the care of various doctors and consulting many more, until they advised him that amputation of the diseased member was his only hope.

It is most painful, under any circumstances, to lose an arm, but much more so to one who is dependent on manual labor for the support of himself and family. Hence our patient preferred death rather than prove a helpless burden on his family by thus living useless and mutilated. Holy resignation was rewarded in a way beyond all expectations.

A carter by trade, he made frequent trips to the suburbs of this city, thus sustaining his unhappy wife. In one of these many journeys he was advised to consult me, which he did, hoping only that I would sustain him in his decision.

Thin, pale, debilitated by suppuration, and fearing I would reiterate the decision that had been made by other doctors at distinct times, all of which placed him in a deplorable moral state, he came to me for the first time, ten years after he had received the wound that caused his later sufferings. The fore-arm without any movement, hollow in the muscular interspaces, presented two solutions of continuity; two fistulous tracks, one in the superior internal part, a centimetre and a half below the olecranon process, and the other in the inferior part at the same point before mentioned. Flowing from both orifices was bad conditioned, sanguineous pus. Introducing the probe from one extreme to the other of the track, I noted with ease the existence of the bone deprived of the periosteum ; in passing the probe it was stopped by the bony roughness, which proved that the tables of the bone were destroyed to a great extent. There was very little pain when remaining quiet in any position, but very sensitive to movement. He always carried it tightly bound, uncovering only when a change of application was necessary.

I proceeded to give him moral courage, kind counsel, and healthy reflections, such as was needed by so unfortunate a father of a family. I assured him that he would not lose his arm, but, on the contrary, it could be saved so that he could dedicate himself to his family's support by his work. This consolation so comforted him that without doubt it contributed to his cure.

I resolved to give him sixteen globules of Phos.$^{30}$, dissolved in eight spoonfuls of water to take every six hours. This prescription was repeated three times.

After eight days I saw him again. His emaciated face and dying look were reanimated by hope, and his eyes had a pleased and happy expression. The suppuration continued, but presented a better character, and his appetite, previously lost, had reappeared, the dominant fever ceasing. He continued using the medicine for several days till the pus became sanious. I then gave him some doses of Hepar-sulph. with the object of obtaining the dislodgment of the enormous sequestra. Under its action the inferior opening increased its dimensions to two centimetres or a little more, the bone acquired more mobility, and the loosened inferior extremity of the necrosed sequestrum appeared at the fistulous opening.

He continued the same for twenty days, then without any aid he drew away the sequestrum of fifteen centimetres and a half without pain, without hemorrhage, and without trouble.

Silicea for four days completed the cure.

To-day Miguel works as if nothing had ever disturbed his arm, loads and unloads his cart with ease, and, thanks to the virtue of the infinitesimal globules, he is not a burden to his family, but rather a help and support.

Does this say anything in favor of Homœopathy? If not sufficient I will relate another case, very distinct, that can never be attributed to chance.

Mrs. J. F., age twenty-one years, living at Vilaseca, married, of good constitution, appeared at my office, January 4th, 1889, together with her young and affectionate husband, justly alarmed by the grave prognostications of several doctors who had examined her for a complaint from which she had suffered for seven months before. It had arrived at such an extreme that she had been advised to go to Barcelona to be operated on, and before undergoing the operation to confess herself and make her will.

Such advice to a young and happy woman was not cheering.

As a last resort she determined to try Homœopathy, and but a few days passed before she repented of not having done so from the first day.

Let us examine the symptoms of this patient.

Of apparently good exterior appearance, all excepting a paleness of face, and slight lividness of the lips, indicated her sufferings. The abdomen was much swollen, having the appearance of a woman in the last stages of pregnancy. Of this she complained. I examined her at once, and discovered a large quantity of fluid in the abdomen which was of globular form, more prominent above the umbilical and epigastric regions. On slight percussion the fluxion was noticeable, the slight pain was increased by the weighty feeling. The respiration impaired as a result of mechanical distention, it was rather dyspnœa, compelling the patient to seek a semi-recumbent position. There were frequent, although not continuous, palpitations, some vomiting and impaired digestion, and the tendency to lipoma constituted the general condition of our patient. Let us add, menstruation normal, the menstrual hemorrhage appearing several days before the regular time. No cause could be attributed to this as she had not taken cold, suppressed any perspiration, or exposed herself to any dampness.

Diagnosis : Ordinary dropsy. I gave one dose of Sulphur, then six of Apocyn-cannab.[3] to dissolve in eight spoonfuls of water, each containing a quarter of a drop, to be taken every three hours.

The 26th of same month I again saw the patient. The improvement was remarkable. The abdominal effusion was diminished to about two-thirds, the only complication was a slight constipation, that was relieved by a simple enema (cold water). This was ultimately cured by some globules of Nux-vom., returning to the Apocyn-cannab. in same proportions as before, and on the 15th of February she was pronounced cured. Two months later she had a relapse, but with prompt treatment it disappeared within a few days, and up to date she is well. How can a few drops of a dilution where there is no medicinal properties (according to the allopaths) overcome a case of dropsy that could not be cured, but only alleviated by operation ?

Let us take another case. E. A., a lovely girl from a neighboring town, consulted me for fatigue that she experienced on the slightest exercise. Menstruation normal: slightly scarce, good constitution, a healthy color. Suffering from slight cephalalgia at times, and a light dry cough, this was all. On examination of the præcordial region, where she complained of a slight pain, some palpitation was apparent. Auscultation revealed an exaggerated movement of the heart, both in strength and frequency. Family history good, she had never experienced trouble, frights, fatigues, or emotions. There were no good indications to account for the diseased state.

At once I judged it to be a nervous affection, and prescribed Hydrocyanic acid. Several days passed with no relief. Cactus gr. gave no better result, the same was true of other medicines. When I had almost despaired of ever curing an affection which, at first, had appeared so trifling, while talking about her case to an illustrious friend and great admirer of Homœopathy, he alluded to her moral character. Both her family's good reputation as well as her own had placed her above doubt, her age and suspicious signs in her physiognomy which I now noticed led me to suspect self-abuse, and that the palpitation originated from this cause. I questioned her in a discreet way, and in effect my suspicions were verified. Tarent.[6] in a few days, caused the complaint to disappear. Also this exaggerated condition of nymphomania, with the cephalalgia, cough, palpitation, and existing fatigue.

Is this cure also chance? How, in this case, were some medicines fallible and others not? Precisely because the applications were wrong, and did not correspond to the especial symptoms that the patient did not confess. As soon as Tarent. was used—that was the corresponding remedy—the disease disappeared and health was restored.

After all, allopathic gentlemen, is not this sufficient proof for you who, with foolish disdain, have decided that "homœopathic dilutions do not contain medicine, and cures obtained by them are only owing to chance."

